# Path analysis of phenotypic traits in young cacao plants under drought conditions

**DOI:** 10.1371/journal.pone.0191847

**Published:** 2018-02-06

**Authors:** Emerson Alves dos Santos, Alex-Alan Furtado de Almeida, Marcia Christina da Silva Branco, Ivanildes Conceição dos Santos, Dario Ahnert, Virupax C. Baligar, Raúl René Valle

**Affiliations:** 1 Departamento de Ciências Biológicas, Universidade Estadual de Santa Cruz, Campus Soane Nazaré de Andrade, Rod, Jorge Amado, Ilhéus, BA, Brasil; 2 United States Department of Agriculture, Agricultural Research Service, Beltsville, MD, United States of America; 3 Centro de Pesquisas do Cacau, Comissão Executiva do Plano da Lavoura Cacaueira (CEPEC/CEPLAC), Rod, Jorge Amado, Ilhéus, BA, Brasil; Estacion Experimental del Zaidin, SPAIN

## Abstract

Drought is worldwide considered one of the most limiting factors of *Theobroma cacao* production, which can be intensified by global climate changes. In this study, we aimed to investigate the phenotypic correlation among morphological characteristics of cacao progenies submitted to irrigation and drought conditions and their partitions into direct and indirect effects. Path analysis with phenotypic plasticity index was used as criteria for estimation of basic and explanatory variables. The experiment was conducted in a greenhouse at the Cacao Research Center (CEPEC), Ilhéus, Bahia, Brazil, in a randomized block 21 x 2 factorial arrangement [21 cacao progenies obtained from complete diallel crosses and two water regimes (control and drought)] and six replications. In general, drought conditions influenced biomass production in most progenies, causing significant reductions in total leaf area, leaf number, leaf biomass, fine-roots length (diameter <1 mm), root volume and root area for considered drought intolerant. All progenies showed alterations in growth due to drought. Phenotypic plasticity was most strongly pronounced in root volume. Stem and root diameters, as well as stem dry biomass were the growth variables with the greatest direct effects on root volume under drought conditions, these characters being indicated in screening of cacao progenies drought tolerant.

## Introduction

Climate oscillations, especially those related to quantity and distribution of rainfall, have been a major cause of variation in cacao (*Theobroma cacao* L.) productivity [1, 2]. Cacao is considered a very sensitive to drought species and can be greatly affected by climate changes and water availability [3, 4]. However, few studies have been conducted to identify cacao adaptation strategies to water deficit [5, 6, 2] and eventual use of water under drought conditions [7]. Some authors have described morphological alterations as indicators for early selection of drought tolerant genotypes [8, 9, 10] as they have a direct impact patterns of plant growth and development [11, 12].

In breeding programs, knowledge of the genetic relationship between traits is important because it allows the breeder to know how the selection for a character may induce simultaneous changes in other traits [13]. The relationship between and among traits can be explained by pleiotropic effects. Moreover, genes closely linked within the same chromosome are also important, especially in populations derived from wide crosses. If two traits have significant genetic relationship, it is possible to obtain gains for one of them, by indirect selection. In some cases, the indirect selection based on the correlated response may produce much more than direct selection for a desired trait. Therefore, indirect selection can be done by selecting a trait of high heritability strongly related to a desired trait [14]

The degree of association between traits is obtained by measuring the value of the correlation coefficient between phenotypic characteristics. These characteristics are of genetic and environmental nature, however, only those of genetic origin should be used in breeding programs [15, 16]. Despite the correlation coefficient is widely used in quantifying factors influence in the determination of complex traits, there is no exploration of the relative importance of direct and indirect effects of these factors [17]. The quantification and interpretation of the magnitude of a correlation can, however, result in errors in the selection strategy because a high correlation between two traits may be the result of effects of a third trait or a group of traits [15] and does not determine the direct and indirect influences between them [18].

To better understand the causes of association among traits, Wright [19] proposed a method called path analysis. In this method, there are quantifications of the direct and indirect effects of the explanatory variables on one basic variable from path coefficients obtained by regression equations [20,15]. The construction of the path diagram showing interrelation between characters, according to the hypotheses to be tested, allows visualization of the direct effect that a character causes in another and the indirect effects of other related characters [16], helping the breeder to decide on the use of correlated responses or of selection indices in breeding programs.

Path analysis has been used by several authors in economically important crops such as passion fruit [21], canola [22], wheat [23], cacao [24] and soybeans [18]. Despite their applicability, the estimated path coefficients may be affected by the effects of multicollinearity [22], caused when there is some level of interrelation between variables. In the presence of multicollinearity, the variances associated with the estimators of the path coefficients can reach too high values, making them unreliable, with no coherence with the biological phenomenon studied [23]. To avoid these effects, it is of fundamental importance to test the degree of collinearity between independent variables [22].

In this study, we aimed to investigate the phenotypic correlation among morphological characteristics of cacao progenies submitted to irrigation and drought conditions, and their partitions into direct and indirect effects through path analysis with phenotypic plasticity index as criteria for estimation of basic and explanatory variables. We consider the hypothesis that morphological alterations play an important role in tolerance of cacao progenies to water stress and that at least one morphological character has potential for direct or indirect selection of drought tolerance.

## Materials and methods

### Genetic material and experimental procedures

Seven cacao accessions chosen from the Active Germplasm Bank of the Cacao Research Center, main research unit of the Commission for the Cacao Farming Plan (CEPEC/CEPLAC), were used in this study for progenies generation (Table 1). No specific permission was required, since the greenhouse studies did not involve endangered or protected species. The selected accessions are used as parents in cacao breeding programs in Brazil and were crossed with each other in a diallel scheme using manual pollination [25]. Recently, it was found that progenies resulting from these crosses show different levels of drought tolerance [26].

**Table 1:** Cacao genotypes used in the diallel crosses and its main characteristics.

Genotype	Origen	Leaf	Flower (N° ovules)		Diseases	
				Pod Index (pods kg^-1^)	Witches’ Broom	*Ceratocystis* Wilt
SCA-6	Peru	L: 236 mm W: 70 mm	42	47	R	S
CATONGO	Brazil	L: 293 mm W: 113 mm	37	23	-	S
MOCORONGO	Brazil	-	-	28	-	S
PUCALA	Peru	-	-	23	-	-
IMC-67	Peru	L: 300 mm W: 91 mm	48	22	S	S
TSH-1188	Trinidad Tobago	L: 236 mm W: 70 mm	56	18	R	R
RB-40	Brazil	-	50	-	R	-

**Source:** International Cocoa Germplasm Database, 2015.

L–length; W—width; R–Resistant; S–Susceptible

In total, 21 progenies were tested. From each progeny, 60 seeds were randomly chosen and planted in 25 L plastic pots containing soil as substrate. The soil was analyzed for its physical and chemical characteristics and the results used to fertilize seedlings, according to the requirements of the cacao crop [27]. The experiment was conducted in a greenhouse of CEPEC, in Ilhéus, Bahia, Brazil (14° 47'S, 39° 16'W), during 2011–2012.

At 12 months after planting, the obtained progenies were divided into two groups: (i) one group with 126 plants (6 replications per progeny) was subjected to water deficit. The water deficit was obtained by gradually reducing the water content of the soil by withholding water up to 60 days, until the predawn leaf water potential (Ψ_WL_) reached –2.0 to –2.5 MPa; (ii) one group with 126 plants (6 replications per progeny) was used as control, receiving daily irrigation to maintain soil moisture close to field capacity with Ψ_WL_ values between –0.1 to –0.5 MPa, totaling 252 plants.

### Predawn leaf water potential (Ψ_WL_)

The Ψ_WL_ measurements were performed in the second or third mature leaf from the apex of the orthotropic axis between 02:00 and 04:00 h using a PMS Model 1000 pressure chamber (PMS Instrument Company, USA) according to methodology described by Scholander [28].

### Growth parameters

Sampling of plant material was done 12–14 months after planting. To determine the beginning of water stress caused by irrigation suspension, Ψ_WL_ measurements were taken daily. Thus, between 20 to 60 days due to differences in progenies drought tolerance, control and drought samples were collected simultaneously. Immediately after sampling, when Ψ_WL_ reached –2.0 to –2.5 MPa in plots under drought conditions, plants were removed from pots and divided into root, stem and leaves.

In both groups of plants, measurements of total (TLA) and individual (ILA) leaf area, stem diameter (SD), plant height (H) and plant leaf number (LN) were performed. TLA was measured with LI-3100 leaf area meter (LI-COR Biosciences, Lincoln, Nebraska, USA), while SD and H were measured using digital calipers and ruler, respectively. The roots were washed 3x with demineralized water, placed in white plastic trays containing between 1.0–2.0 cm depth water lamina. After submersion in a water bath to facilitate root separation of roots and to minimize overlap when photographed (Sony Lens 4x optical 12.1 Megapix). Soon after, roots, stems and leaves from each progeny were stored separately in paper bags and dried in a forced-air oven at 75°C until constant mass weight. From the samples dry root (RB), stem (SB), leaf (LB) and total (TB) biomasses as well as root/shoot ratio (R/S—RB/(SB + LB) were estimated.

Later, images of the plant root system were digitized in the Integrated System for Roots and Land Cover Analysis using the WinRhizo software, version 2013 (Regent Instrument, Quebec, Canada), which was calibrated to obtain total root length and average root diameter based in earlier known area.

After image processing, values for root area (RA), root length (RL), average root diameter (RD) and root volume (RV) were obtained.

Roots length were assigned to ten diameter classes: five were used between 0 and 2 mm (fine and medium roots length) and five diameter classes bigger than 2 mm (coarse roots length). Estimation of roots classes was based on cacao fine roots studies of cacao fine roots described previously by Kummerow et al. [29].

### Statistical analysis

The data were analyzed as a randomized block design considering 42 treatments [21 progenies x 2 water systems (control: Ψ_WL_ between –0.1 and –0.5 MPa and stressed: Ψ_WL_ between –2.0 and –2.5 MPa)] and 6 replications. The average of each progeny in different water regimes were compared with the overall average of each growth variable using Student’s t test (p < 0.05 and p < 0.01).

Later, the 17 growth variables (H, SD, TLA, LN, ILA, RB, LB, SB, TB, R/S, RL, RA, RD, RV, RD[<1mm], RD[1-2mm] and RD [>2mm] were standardized due to differences in units. Standardization was based on the equation: Z_ij_ = (X_ij_−X_j_)/S_j_, where X_ij_ is the value of i^th^-observation of variable X_j_; X_j_ and S_j_ are the mean and standard deviation of X_j_, respectively.

All standardized variables were subjected to factorial analysis, using the computing environment R, version 3.03 for Windows [30]. The results showed that only 13 variables were relevant for the formation of the first three factors (total variation 70%). These variables were then submitted to collinearity analysis based on tolerance and the variance inflation factor (VIF), considering as threshold for inclusion, a value greater than 0.1 and less than 10, respectively [31].

The 11 variables considered no collinear (SD, TLA, LB, SB, RB, TB, RL, RD, RV, RD[< 1 mm] and RD[1-2mm] were used for estimation of the phenotypic plasticity index (IPF) as Valladares [32, 33], where: IPF = (Max-Min) / Max. The IPF values were calculated based on the average of the most contrasting treatments, being the maximum value of the control and the minimum of the stressed. The data obtained from IPF were used for selecting the basic variable and the explanatory variables based on Scott & Knott test (p < 0.05 and p <0.01).

Phenotypic correlations between characters were estimated from the individual values according to the equations:

$$r_{r_{F_{XY}}}=\frac{\sigma_{F_{X}F_{Y}}}{\sqrt{\sigma^{2}F_{X}\cdot \sigma^{2}F_{Y}}}$$

Where $r_{F_{XY}}$ is phenotypic coefficients of variation, and *σ_Fx Fy_* the phenotypic cross-products of characters *x* and *y*, estimated from covariance analysis; σ^2^_*Fx*_σ^2^_*Fy*_ is the phenotypic variances of characters *x* and *y*, respectively.

It was tested the significance of all phenotypic correlation coefficients by the t test, with n-2 degrees of freedom at 5% and 1% significance levels as follows:

$$t=\frac{r}{\sqrt{1-r^{2}}}\sqrt{n-2}$$

where r = the estimated correlation coefficient; and n = number of plants assessed.

Regression analysis was used to quantify the relationship between root diameter and root length. Path analysis was performed according to a causal diagram of two chains, using GENES software [34].

## Results

### Growth parameters

Soil water deficit influenced (<p 0,05) biomass the plants, except for H, ILA, SB, TB, RL, RA and RD[1- 2mm] (Table 2).

**Table 2:** Average means of growth variables (μ ± SE) and absolute deviations (Δ) for single cacao progenies, obtained from complete diallel crosses, under control (predawn Ψ_WL_ between -0.1 and -0.5 MPa) and drought (predawn Ψ_WL_ between –2.0 to –2.5 MPa) conditions. H = plant height (m); SD = stem diameter (mm); LN = leaves number per plant; TLA = total leaf area per plant (m^2^ x10^-4^); ILA = individual leaf area (m^2^ x 10^−2^); RB = root biomass (g); SB = stem dry biomass (g); LB = leaf dry biomass (g); TB = total dry biomass (g); R/S = root:shoot ratio; RL = root length (mm); RA = root system area (cm^2^); RD = root mean diameter for second order branches (mm); RV = root volume (cm^3^); RD[<1 mm] = fine roots length (cm cm^-3^); RD[1-2mm] = medium roots length (cm cm^-3^); RD[>2mm] = coarse roots length (cm cm^-3^). (1) = RB-40 x SCA-6; (2) = RB-40 x IMC-67; (3) = RB-40 x Catongo; (4) = MOC-01 x IMC-67; (5) = SCA-6 x IMC-67; (6) = IMC-67 x TSH-1188; (7) = MOC-01 x SCA-6; (8) = PUCALA x MOC-01; (9) = Catongo x TSH-1188; (10) = TSH-1188 x SCA-6; (11) = MOC-01 x TSH-1188; (12) = Catongo x IMC-67; (13) = MOC-01 x Catongo; (14) = PUCALA x Catongo; (15) = PUCALA x TSH-1188; (16) = RB-40 x MOC-01; (17) = Catongo x SCA-6; (18) = PUCALA x RB-40; (19) = PUCALA x SCA-6; (20) = PUCALA x IMC-67; (21) = RB-40 x TSH-1188.

Variable	WR	μ ± SE	Diallel crosses (Δ)																				
			1	2	3	4	5	6	7	8	9	10	11	12	13	14	15	16	17	18	19	20	21
H	C	1.9±0^a^	0^ns^	0^ns^	0^ns^	0^ns^	0^ns^	0^ns^	0^ns^	0^ns^	0^ns^	0^ns^	0*	0^ns^	0^ns^	0^ns^	-0^ns^	0^ns^	0^ns^	-0*	0*	-0*	0^ns^
	D	1.8±0^a^	0*	0^ns^	0^ns^	0^ns^	0*	0^ns^	0*	-0^ns^	0^ns^	0^ns^	-0***	0^ns^	-0***	-0***	0^ns^	0^ns^	0*	0^ns^	-0^ns^	-0**	0^ns^
SD	C	21±1^a^	4*	-1^ns^	-1^ns^	0^ns^	2^ns^	0^ns^	-0^ns^	-2^ns^	-4**	-1^ns^	-0^ns^	-1^ns^	3**	0^ns^	0^ns^	2*	3**	-2^ns^	0^ns^	-3**	-0^ns^
	D	19±1^b^	1^ns^	-1^ns^	2^ns^	-2*	4***	-1^ns^	-2^ns^	0^ns^	-2*	1^ns^	-2*	1^ns^	2^ns^	-3***	-3***	2*	1^ns^	0^ns^	-1^ns^	0^ns^	0^ns^
LN	C	61±5^a^	2^ns^	3^ns^	-2^ns^	-2^ns^	4^ns^	18***	7^ns^	2^ns^	-9*	20***	20***	-14*	-3^ns^	-1^ns^	0^ns^	-8^ns^	1^ns^	-16**	-11*	-18***	7^ns^
	D	46±5^b^	9^ns^	0^ns^	-4^ns^	0^ns^	7^ns^	16***	13*	-7^ns^	1^ns^	5^ns^	18***	-10*	4^ns^	-17***	-1^ns^	-0^ns^	3^ns^	-12***	-15***	-16***	6^ns^
TLA	C	1±0^a^	0^ns^	-0*	-0*	0^ns^	0^ns^	0*	0^ns^	0^ns^	0^ns^	0**	0^ns^	0^ns^	0^ns^	0*	-0^ns^	0^ns^	0^ns^	-0**	-0^ns^	-0**	0^ns^
	D	0±0^b^	0^ns^	0*	0^ns^	0^ns^	0*	0^ns^	0^ns^	0^ns^	0***	-0***	0^ns^	0^ns^	0^ns^	-0**	-0^ns^	0^ns^	0^ns^	-0^ns^	-0^ns^	-0***	0^ns^
ILA	C	2±0^a^	0^ns^	-0***	-0***	-0^ns^	-0^ns^	0^ns^	0^ns^	-0^ns^	0^ns^	-0^ns^	-0***	0***	0^ns^	0***	-0***	0***	-0^ns^	0^ns^	0*	0^ns^	-0^ns^
	D	2±0^a^	1^ns^	2^ns^	2^ns^	1^ns^	2^ns^	1^ns^	1*	2^ns^	2^ns^	1***	1***	2*	1^ns^	2***	1***	2***	2^ns^	2***	2^ns^	1*	2^ns^
RB	C	29±3^b^	-1^ns^	-0^ns^	2^ns^	2^ns^	-6*	9**	5*	-1^ns^	1^ns^	1^ns^	4^ns^	-6***	-5**	-5*	3^ns^	2^ns^	0^ns^	-3^ns^	-4*	-0^ns^	2^ns^
	D	34±3^a^	-0^ns^	2^ns^	-0^ns^	3^ns^	1^ns^	-5*	2^ns^	-1^ns^	-6*	-2^ns^	-2^ns^	4^ns^	18***	-4*	2^ns^	5*	2^ns^	-2^ns^	-9**	-3^ns^	-4^ns^
SB	C	68±7^a^	12^ns^	-2^ns^	-2^ns^	-0^ns^	4^ns^	24***	11^ns^	-5^ns^	-21***	-1^ns^	13*	1^ns^	24***	-14*	-1^ns^	11^ns^	10^ns^	-25***	1^ns^	-30***	-8^ns^
	D	67± 7^a^	22**	-1^ns^	4^ns^	-5^ns^	29***	-6^ns^	-15*	-4^ns^	-3^ns^	-2^ns^	-5^ns^	17**	8^ns^	-27***	-10^ns^	29***	9^ns^	-1^ns^	-15*	-30***	7^ns^
LB	C	66±4^a^	11^ns^	-3^ns^	-11^ns^	-4^ns^	13*	4^ns^	3^ns^	0^ns^	-15^ns^	-0^ns^	3^ns^	-1^ns^	20**	3^ns^	-21***	4^ns^	19***	-18***	-3^ns^	-10^ns^	5^ns^
	D	53±3^b^	10*	0^ns^	-3^ns^	-3^ns^	18***	-2^ns^	7^ns^	-1^ns^	4^ns^	-16^ns^	-5^ns^	-1^ns^	17***	-21***	-11*	17***	2^ns^	-1^ns^	-15**	0^ns^	5^ns^
TB	C	173±15^a^	40*	-14^ns^	-16^ns^	5^ns^	50***	11^ns^	3^ns^	-0^ns^	-42**	-10^ns^	7^ns^	-12^ns^	70***	23^ns^	-37**	12^ns^	20^ns^	-58***	-2^ns^	-42**	-6.6^ns^
	D	159±15^a^	30*	-16^ns^	13^ns^	-18^ns^	63***	-4^ns^	-5^ns^	-6^ns^	-10^ns^	-4^ns^	-28*	2^ns^	43***	-58***	-16^ns^	21^ns^	10^ns^	-7^ns^	-9^ns^	-29*	8^ns^
R/S	C	0.2±0.0^b^	0***	0^ns^	0^ns^	0^ns^	0^ns^	0^ns^	0^ns^	0^ns^	0**	0^ns^	0^ns^	0*	0^ns^	0^ns^	0^ns^	0^ns^	0^ns^	0**	0^ns^	0^ns^	0^ns^
	D	0.3± 0.0^a^	-0**	0^ns^	0^ns^	0^ns^	0*	0^ns^	-0***	0^ns^	-0**	0^ns^	0^ns^	0***	0*	0^ns^	0^ns^	0^ns^	0^ns^	0^ns^	0^ns^	0^ns^	0.^ns^
RL	C	3501±103^a^	35^ns^	-11^ns^	47^ns^	47^ns^	53^ns^	52^ns^	31^ns^	31^ns^	-28^ns^	85*	38^ns^	89*	-18^ns^	-55^ns^	-116***	50^ns^	4^ns^	-71*	-109***	-175***	20^ns^
	D	3307±160^a^	-43^ns^	9^ns^	12^ns^	-23^ns^	51^ns^	-13^ns^	29^ns^	-4^ns^	-18^ns^	36^ns^	-7^ns^	-29^ns^	23^ns^	-118***	112***	94***	-4^ns^	53^ns^	-13^ns^	-93***	-56*
RA	C	970±36^a^	13^ns^	340*	295^ns^	212^ns^	184***	227^ns^	146^ns^	50^ns^	-241^ns^	325^ns^	69^ns^	341***	8^ns^	-225^ns^	-394**	-15^ns^	-6^ns^	-310*	-70^ns^	-574**	-376**
	D	667±33^a^	-209*	100^ns^	60^ns^	-144^ns^	680***	-138^ns^	-27^ns^	-177^ns^	-82^ns^	174^ns^	174^ns^	103^ns^	-80^ns^	-286^ns^	57^ns^	53^ns^	39^ns^	282^ns^	-197^ns^	-95^ns^	-286*
RD	C	2±0^a^	-0^ns^	0*	0*	0^ns^	1***	0^ns^	0^ns^	-0^ns^	-0^ns^	0^ns^	0^ns^	0^ns^	0^ns^	-0*	0^ns^	-0*	-0^ns^	-0*	-0^ns^	-0^ns^	-0^ns^
	D	1±0^b^	-0*	-0^ns^	0^ns^	-0*	0^ns^	-0*	-0^ns^	-0**	-0^ns^	0*	0^ns^	0**	0^ns^	0^ns^	0^ns^	-0^ns^	0^ns^	0^ns^	-0^ns^	0^ns^	-0*
RV	C	234±10^a^	-28^ns^	129**	78*	74^ns^	224***	60^ns^	89^ns^	-41^ns^	-76^ns^	121*	-5^ns^	28^ns^	-17^ns^	-107*	-53^ns^	-67^ns^	-36^ns^	-123**	-26^ns^	-147**	-73^ns^
	D	104±24^b^	-47^ns^	-35^ns^	25^ns^	-34^ns^	116***	-36^ns^	-14^ns^	-47*	-17^ns^	48**	-18^ns^	67*	-13^ns^	-30^ns^	32^ns^	18^ns^	23^ns^	76**	-45*	-14^ns^	-53*
RD[<1 mm]	C	1588±146^b^	347*	-111^ns^	-89^ns^	17^ns^	-94^ns^	294*	-171^ns^	301^ns^	-160^ns^	138^ns^	-115^ns^	423*	-149^ns^	-18^ns^	-346**	310^ns^	-20^ns^	-224^ns^	-222^ns^	-315**	206^ns^
	D	1864±124^a^	-59^ns^	162^ns^	-86^ns^	-109^ns^	545***	91^ns^	144^ns^	355*	-118^ns^	-92^ns^	-41^ns^	-260^ns^	25^ns^	-517****	206^ns^	263^ns^	-132^ns^	37^ns^	-49^ns^	-216^ns^	-146^ns^
RD[1-2mm]	C	970±120^a^	129^ns^	-227*	148^ns^	34^ns^	29^ns^	130^ns^	-68^ns^	337**	-117^ns^	263*	-48^ns^	39^ns^	-227*	-18^ns^	-386***	338***	-29^ns^	-98^ns^	-125^ns^	-409***	304***
	D	929±161^a^	-158^ns^	329*	-2^ns^	-65^ns^	420**	-133^ns^	-159^ns^	39^ns^	-50^ns^	128^ns^	88^ns^	-73^ns^	13^ns^	-460***	363**	163^ns^	-51^ns^	109^ns^	-60^ns^	-176^ns^	-265^ns^
RD[>2 mm]	C	1143±191^a^	-303*	25^ns^	276^ns^	72^ns^	515***	271^ns^	302^ns^	187^ns^	-333*	506*	39^ns^	294^ns^	-102^ns^	-323*	-395*	-109^ns^	-92^ns^	-417***	-68^ns^	-348*	0^ns^
	D	706±149^b^	-254*	77^ns^	193^ns^	-188*	535***	-217*	46^ns^	-204^ns^	-103^ns^	188^ns^	178^ns^	-19^ns^	73^ns^	-254*	264*	-90^ns^	88^ns^	342*	-248*	-78^ns^	-330**

Statistical significance between the overall averages of each condition (control and drought) for each growth variable was analyzed by Student's t-test; averages followed by the same letter do not statistically differ at 5% probability. Statistical significance of the difference (Δ) between the mean of each progeny and the overall average, for each condition (control and drought) and growth variable, was analyzed by Student's t-test

(*) p < 0.05

(**) p< 0.01

(***) p < 0.001

(ns) non–significant

Regarding plant height (H) and stem diameter (SD), under drought conditions, only progenies originated from MOC-01 x TSH-1188 (11) and PUCALA x Catongo (14) crosses showed significant reductions of their averages, with values below the overall average. Under the same conditions, progenies of SCA-6 x IMC-67 (5) and RB-40 x MOC-01 (16) showed values above the expected average.

Reduction in the leaf number (LN) and of total leaf area (TLA) was also observed in progenies subjected to soil water limitation. Progenies of RB-40 x IMC-67 (2), SCA-6 x IMC-67 (5) and Catongo x TSH-1188 (9) showed values above the overall average. In contrast, TSH-1188 x SCA-6 (10), PUCALA x Catongo (14) and PUCALA x IMC-67 (20) showed significant reductions in leaf area when subjected to soil water deficit.

The decrease of leaf area (TLA) had a direct impact in the leaf biomass (LB) of cacao progenies submitted to soil water deficit, reducing the overall average by almost 20% when compared to the control (Table 2). However, progenies of RB-40 x SCA-6 (1), SCA-6 x IMC-67 (5), MOC-01 x Catongo (13) and RB-40 x MOC-01 (16) showed significant increases in LB when compared to the overall average under drought conditions.

Progenies of RB-40 x SCA-6 (1), SCA-6 X IMC-67 (5), Catongo X IMC-67 (12) and RB-40 x MOC-01 (16) showed increment in stem biomass (SB) above the overall average under limiting soil water conditions. In contrast, progenies of MOC-01 x SCA-6 (7), PUCALA x Catongo (14), PUCALA x SCA-6 (19) and PUCALA x IMC-67 (20) showed averages below the overall average.

Under water limitation, the increase in root biomass was higher than the control, with effects in RB, R/S, RA and RV (Table 2). The progenies of MOC-01 x Catongo (13), SCA-6 x IMC-67 (5) and Catongo x IMC-67 (12) showed significant root biomass increase compared to the overall average. In contrast, IMC-67 x TSH-1188 (6), Catongo x TSH-1188 (9) and PUCALA x Catongo (14) progenies showed lower averages when compared with the overall average.

**Fig 1:**
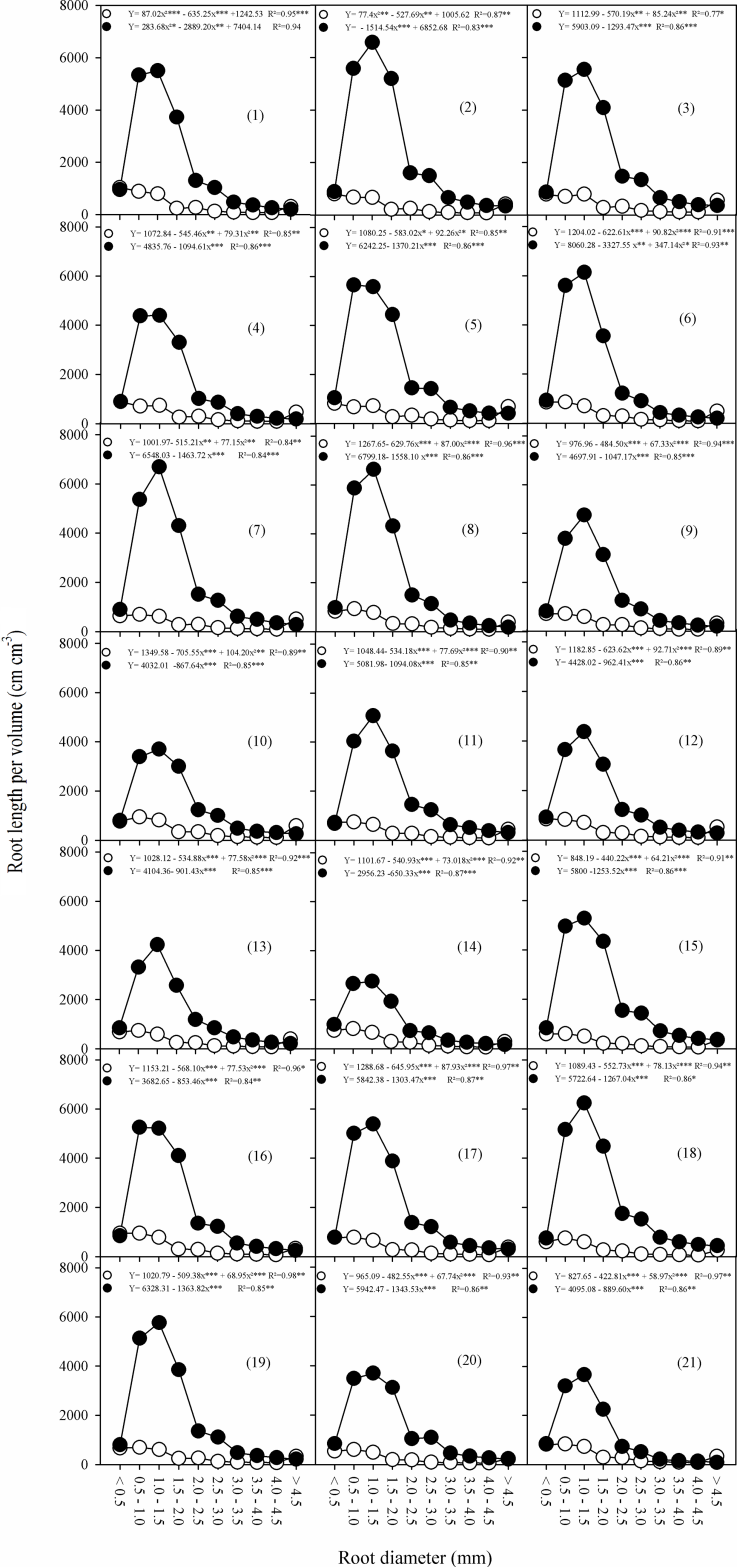
Root length per volume of cacao progenies, obtained from complete diallel crosses, under control (○) (predawn Ψ_WL_ between -0.1 and -0.5 MPa.) and drought (●) (predawn Ψ_WL_ between -2.0 and -2.5 MPa) conditions.

Regarding fine-roots length RD[<1mm], comparing the overall mean of the variable with the average of each progeny it was found that SCA-6 x IMC-67 (5), PUCALA x MOC-01 (8) and PUCALA x Catongo (14) progenies showed a significant increase of fine root values under drought stress (Table 2). Also, comparing the overall average of the medium-roots length RD[1-2mm] values with the mean of each progeny, only RB 40 x IMC-67 (2), SCA-6 x IMC-67 (5) and PUCALA x TSH-1188 (15) showed significant values increases. For coarse-roots length RD[>2mm], by comparing the overall average of the variable with the mean of each progeny, SCA-6 x IMC-67 (5), PUCALA x TSH-1188 (15) and PUCALA x RB-40 (18) progenies showed a significant increase in water limiting conditions. Most progenies showed a tendency of a progressive increase in fine (RD[<1mm]) and medium (RD[1-2mm]) roots as the soil water limitation increased (Fig 1).

### Path analysis

**Fig 2:**
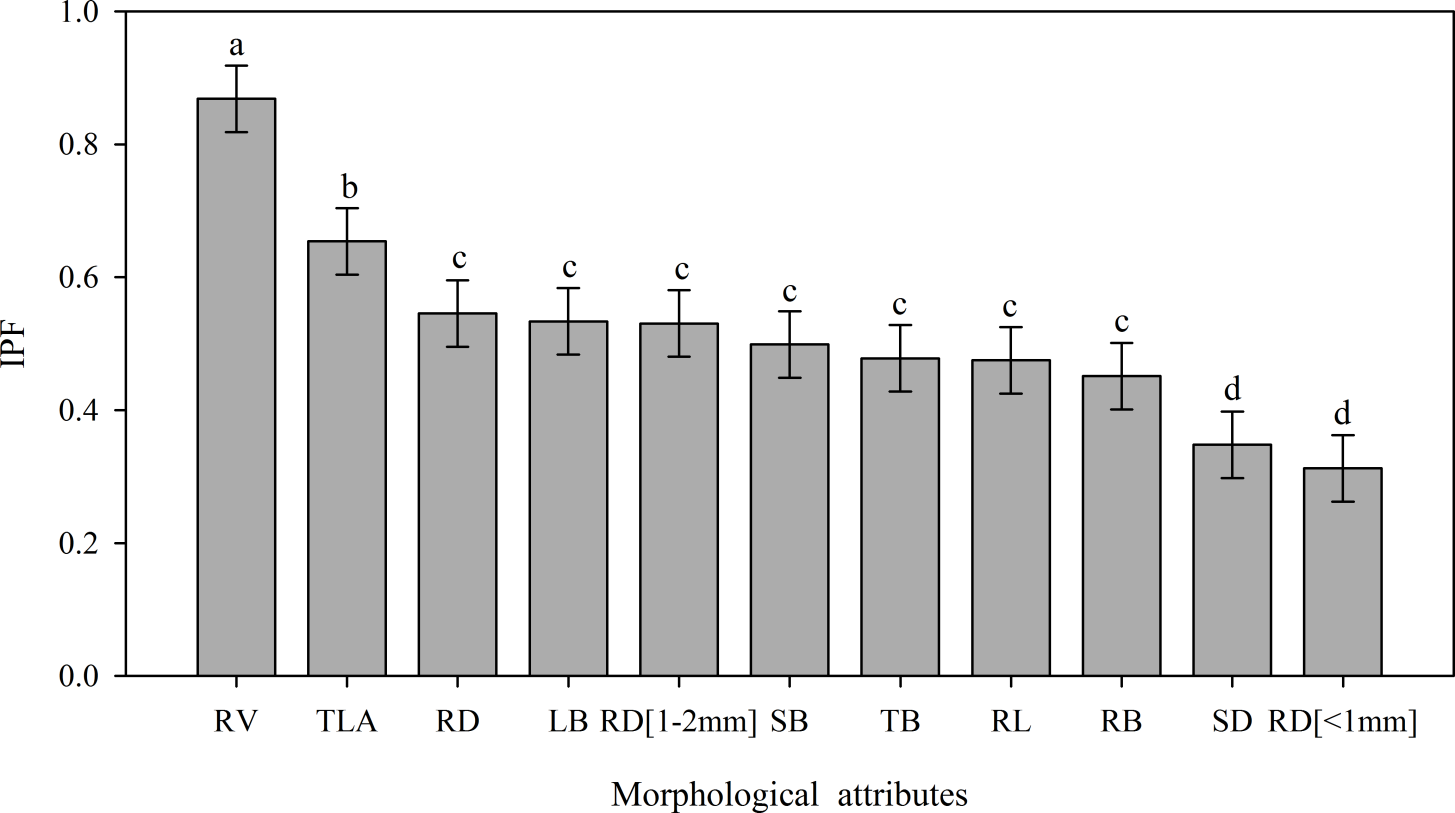
Phenotypic plasticity index (IPF) for growth characters of cacao progenies, obtained from complete diallel crosses, under control (predawn Ψ_WL_ between -0.1 and -0.5 MPa) and drought (predawn Ψ_WL_ between –2.0 to –2.5 MPa) conditions. SD = stem diameter (mm); TLA x 10^−4^ = total leaf area per plant (m^2^ plant^-1^); RB = root biomass (g); SB = stem dry biomass (g); LB = leaf dry biomass (g); TB = total dry biomass (g); RL = root length (mm); RD = root mean diameter for second order branches (mm); RV = root volume (cm^3^); RD[<1 mm] = fine roots length (cm cm^-3^); RD[1-2mm] = medium roots length (cm cm^-3^).

Most morphological characteristics showed variations under the two imposed water regimes (control: Ψ_WL_ between –0.1 and –0.5 MPa; and drought: Ψ_WL_ between –2.0 and –2.5 MPa), demonstrating different schemes of phenotypic plasticity in response to drought. In this study, RV showed the highest IPF value (0.86), which points out the high plasticity in response to drought (Fig 2)

The stem diameter (SD) was positively correlated with RB (0.66**), SB (0.74**), LB (0.77**) and TB (0.82**) under control condition (Table 3). Under drought, SD was additionally related to RV (0.46*) (Table 4). Total leaf area (TLA) was positively correlated with SB, LB, TB and RD[<1mm], both under control and drought conditions. Stem biomass (SB) was positively correlated with LB (0.68**), TB (0.73**), RL (0.54*) and RV (0.45*) in the control condition (Table 3). Under soil water limitation, SB was correlated only with LB and TB.

**Table 3:** Correlation matrix for growth characters of cacao progenies, obtained from complete diallel crosses, under control condition (predawn Ψ_WL_ between -0.1 and -0.5 MPa). SD = stem diameter (mm); TLA = total leaf area per plant (m^2^ x 10^−4^); RB = root biomass (g); SB = stem dry biomass (g); LB = leaf dry biomass (g); TB = total dry biomass (g); RL = root length (mm); RD = root mean diameter for second order branches (mm); RV = root volume (cm^3^); RD[<1 mm] = fine roots length (cm cm^-3^); RD[1-2mm] = medium roots length (cm cm^-3^).

	SD	TLA	RB	SB	LB	TB	RL	RD	RV	RD [<1mm]	RD[1–2 mm]
SD		0.38^ns^	0.66**	0.74**	0.77**	0.82**	0.27^ns^	0.18 ^ns^	0.22 ^ns^	0.23 ^ns^	0.16 ^ns^
TLA			0.43 ^ns^	0.51*	0.57**	0.60**	0.52*	0.04 ^ns^	0.27 ^ns^	0.46*	0.47*
RB				0.51*	0.54*	0.73**	0.08 ^ns^	0.07 ^ns^	0.07 ^ns^	0.07 ^ns^	0.14 ^ns^
SB					0.68**	0.73**	0.54*	0.37 ^ns^	0.45*	0.36 ^ns^	0.24 ^ns^
LB						0.90**	0.44*	0.09 ^ns^	0.26 ^ns^	0.39 ^ns^	0.32 ^ns^
TB							0.41 ^ns^	0.26 ^ns^	0.35 ^ns^	0.31 ^ns^	0.25 ^ns^
RL								0.34 ^ns^	0.61**	0.72**	0.75**
RD									0.89**	-0.18 ^ns^	-0.18 ^ns^
RV										0.11 ^ns^	0.19 ^ns^
RD[<1mm]											0.81**
RD[1-2mm]											

Correlation coefficients comparisons were made by *t*-test

(*) p < 0.05

(**) p < 0.01

(ns) non-significant

**Table 4:** Correlation matrix for growth characters of cacao progenies, obtained from complete diallel crosses, under drought condition (predawn Ψ_WL_ between –2.0 to –2.5 MPa). SD = stem diameter (mm); TLA = total leaf area per plant (m^2^ x 10^−4^); RB = root biomass (g); SB = stem dry biomass (g); LB = leaf dry biomass (g); TB = total dry biomass (g); RL = root length (mm); RD = root mean diameter for second order branches (mm); RV = root volume (cm^3^); RD[<1 mm] = fine roots length (cm cm^-3^); RD[1-2mm] = medium roots length (cm cm^-3^).

	SD	TLA	RB	SB	LB	TB	RL	RD	RV	RD [<1mm]	RD[1–2 mm]
SD		0.13 **	0.59**	0.71**	0.59**	0.80**	0.18 **	0.14 **	0.46*	0.33 **	0.27 **
TLA			0.23 **	0.54*	0.70**	0.48*	0.23 **	-0.37 **	-0.05 **	0.44*	0.22 **
RB			1	0.56**	0.58**	0.62**	0.22 **	0.29 **	0.25 **	0.17 **	0.24 **
SB					0.69**	0.86**	0.41 **	0.08 **	0.42 **	0.42 **	0.40 **
LB						0.76**	0.28 **	-0.22 **	0.16 **	0.50*	0.23 **
TB							0.41 **	0.06 **	0.45*	0.49*	0.37 **
RL								0.09 **	0.52*	0.72**	0.81**
RD									0.74**	-0.30 **	0.15 **
RV										0.28 **	0.55*
RD[<1mm]											0.76**
RD[1-2mm]											

Correlation coefficients comparisons were made by *t*-test

(*) p < 0.05

(**) p < 0.01

(ns) non-significant.

The root volume (RV) was positively correlated with TB (0.45*) under drought conditions (Table 4). The increase in RV was associated with root length (0.52*), especially with medium diameter RD[1-2mm] roots.

A breakdown of phenotypic correlations in direct and indirect effects, through path analysis, indicated that TLA (0.11), LB (0.27), RD[<1mm] (-0,8) and RD[1-2mm] (0,42) showed the largest direct effects on the development of the root system (RV) of the cacao progenies under control conditions (Table 5). On the other hand, in the drought conditions, SD (0.38), LB (0.21), RD (0.93) and RD[<1mm] (0.18) were the major direct effects on the increase of the progenies root volume.

**Table 5:** Path analysis for growth characters of cacao progenies, obtained from complete diallel crosses, under control (predawn Ψ_WL_ between -0.1 and -0.5 MPa).and drought (predawn Ψ_WL_ between –2.0 to –2.5 MPa) conditions. SD = stem diameter (mm); TLA = total leaf area per plant (m^2^ x 10^−4^); RB = root biomass (g); SB = stem dry biomass (g); LB = leaf dry biomass (g); TB = total dry biomass (g); RL = root length (mm); RD = root mean diameter for second order branches (mm); RV = root volume (cm^3^); RD[<1 mm] = fine roots length (cm cm^-3^); RD[1-2mm] = medium roots length- cm cm^-3^).

Variable	Condition	Direct effect	Indirect effect										
		RV	SD	TLA	RB	SB	LB	TB	RL	RD	RD [<1mm]	RD[1-2mm]	Mean
SD	Control	-0.05		0.04	-0.06	0.02	0.21	-0.15	-0.02	0.18	-0.02	0.07	0.22**
	Drought	0.38		0.01	-0.27	-0.06	0.13	0.04	0.05	0.13	0.06	0.01	0.46*
TLA	Control	0.11	-0.02		-0.04	0.01	0.16	-0.11	-0.04	0.04	-0.04	0.2	0.27**
	Drought	0.08	0.05		-0.11	-0.05	0.15	0.02	0.06	-0.35	0.08	0.01	-0.06**
RB	Control	-0.09	-0.03	0.05		0.01	0.15	-0.13	-0.01	0.07	-0.01	0.06	0.07**
	Drought	-0.46	0.22	0.02		-0.05	0.13	0.03	0.06	0.27	0.03	0.01	0.25**
SB	Control	0.03	-0.04	0.06	-0.05		0.19	-0.13	-0.05	0.37	-0.03	0.1	0.45*
	Drought	-0.09	0.27	0.04	-0.26		0.15	0.04	0.11	0.07	0.07	0.01	0.42**
LB	Control	0.27	-0.04	0.06	-0.05	0.02		-0.16	-0.04	0.09	-0.03	0.13	0.26**
	Drought	0.21	0.22	0.06	-0.27	-0.06		0.04	0.07	-0.2	0.09	0.01	0.16**
TB	Control	-0.18	-0.04	0.07	-0.07	0.02	0.25		-0.03	0.26	-0.03	0.11	0.35**
	Drought	0.05	0.3	0.04	-0.29	-0.08	0.16		0.11	0.06	0.09	0.01	0.45*
RL	Control	-0.08	-0.01	0.06	-0.01	0.01	0.12	-0.07		0.34	-0.06	0.32	0.61**
	Drought	0.26	0.07	0.02	-0.1	-0.04	0.06	0.02		0.08	0.13	0.02	0.52*
RD	Control	1	-0.01	0	-0.01	0.01	0.02	-0.05	-0.03		0.01	-0.08	0.89**
	Drought	0.93	0.05	-0.03	-0.13	-0.01	-0.05	0	0.02		-0.05	0	0.74**
RD[<1mm]	Control	-0.08	-0.01	0.05	-0.01	0.01	0.11	-0.06	-0.06	-0.18		0.34	0.11**
	Drought	0.18	0.12	0.04	-0.08	-0.04	0.11	0.02	0.19	-0.28		0.02	0.28**
RD[<2mm]	Control	0.42	-0.01	0.05	-0.01	0.01	0.09	-0.04	-0.06	-0.18	-0.07		0.19**
	Drought	0.02	0.1	0.02	-0.11	-0.04	0.05	0.02	0.21	0.14	0.13		0.55*

Correlation coefficients comparisons were made by *t*-test

(*) p < 0.05

(**) p < 0.01

(ns) non-significant.

The variables SD, RL and RD showed a positive and significant correlation with root volume (RV) under drought conditions. Despite the direct effect of RD[1-2mm] on RV in the drought conditions, the correlation, although positive, was not significant. This may also have been due to the growth of coarse roots (RD[>2mm] (–0.28).

## Discussion

Water stress is one of the factors limiting agricultural production at the global scale [35, 36]. The degree of reversibility of its effects depends duration and severity of drought events, and the genotype and development stage of plants under consideration [37]. Response mechanisms to stress are usually associated with the individual's ability to respond to phenotypic changes to different environmental conditions (phenotypic plasticity). In addition, it appears to be a key mechanism to enable species to respond adaptively to environmental changes [38, 32, 33]

Drought influenced biomass production, reducing the dry weight of all plant organs of most evaluated cacao progenies. Less significant effects were observed on SCA-6 x IMC-67, MOC-01 x Catongo, Catongo x IMC-67 and RB-40 x IMC-67 progenies, which showed average values above the overall average of the crosses both for shoot (H, TLA, SD, LB, SB and LN) and the root system (R/S, RL, RV, RA, RD and RD[<1mm]. On the other hand, progenies of PUCALA x Catongo, PUCALA x MOC-01, IMC-67 x TSH-1188 and MOC-01 x IMC-67 showed a lower tolerance to limited water supply. In comparison to that, studies on *Coffea canephora* also detected differences between drought tolerant and sensitive clones [39, 37]

The decrease in leaf biomass (LB) proved to be an important strategy used by progenies under water limiting conditions (Table 2). When comparing trait values of each progeny with the overall average of the crosses observed that these results were even more evident for PUCALA x SCA-6, PUCALA x Catongo, IMC-67 x TSH-1188 and PUCALA x IMC-67 progenies. Under drought, *Coffea canephora* clones showed leaf fall, sequentially beginning by the older to the younger leaves, suggesting that the higher the clone intolerance to drought, the greater the extent of leaf fall [39]. As the soil dries, water absorption becomes more difficult, which can cause severe plant wilting and subsequently premature leaf dropping.

Progenies Catongo x SCA-6, SCA-6 x IMC-67, MOC-01 x Catongo, RB-40 x MOC-01 and RB-40 x IMC-67 showed trait values related to H, SD, TLA, RB, RV, RD[<1mm] and RD[1-2mm] above the overall average across the sample (Table 2). This can be interpreted as an adaptive response to soil water limitation due to more efficient carbon assimilation [35]. In general, plants limit their growth in response to drought stress. However, genetic variations can provide different plant responses. The water content, derived from stem reserves and supply of assimilates are important strategies that enable plant growth under stress conditions [6].

The root volume (RV) was the growth variable most influenced by the water regimes among the analyzed cacao progenies (Fig 2). These results corroborate with findings in other studies [40, 41]. Although the obvious positive relationship between root growth and yield under drought conditions, the difficulties in evaluating root systems, the large environmental influences and the complex inheritance of root characteristics hinder the use of these traits in selection programmers [38].

Under drought conditions, stem diameter (SD), leaf dry biomass (LB), average root diameter (RD) and fine roots (RD[<1mm] were the growth variables exerting the greatest direct influence on the progenies root volume increase (Table 4). These responses may be associated with different morphological mechanisms that plants use to sustain their growth and development under conditions of low soil water availability. This may involve maximizing root water uptake (dense and deep) or minimizing water loss by stomatal closure and reduction of leaf area [42,43]. These adaptive mechanisms promote, in turn, the improvement in plant water status, particularly in the maintenance of cellular turgor, which can be accomplished through osmotic adjustment or by changes in the cell wall elasticity module [44].

There was a direct positive effect of stem diameter (SD) in root volume (RV) increase under conditions of low soil water availability (Table 5). The effect of drought on stem growth is probably affected by the same factors that limit leaf growth during stress [6]. This effect may be associated with the presence of water content derived from stem reserves and physiological mechanisms of assimilate translocation and stocking in plant compartments during periods of water limitation. This is a desirable feature for genetic improvement of cacao for drought tolerance. However, there is little information on the seasonal dynamics of cacao growth, whose young plants exhibit alternation on growth of shoot and roots [45]. In citrus, soil water deficiency affected negatively vegetative growth [46, 47].

Total leaf area (TLA) showed a negative and not significant correlation with RV under low soil water availability (Tables 3 and 4). Under drought conditions there is a conflict between conservation of water by the plant and the CO_2_ assimilation rate to produce carbohydrates. The need to solve this conflict takes the plant to develop morphophysiological mechanisms that lead to conserve water for use in later periods [48]. However, the reduction of leaf area during water stress [49], is usually accompanied by a decrease in CO_2_ assimilation [50], which can compromise cacao flowering and production [51, 8].

The positive correlation between TB and RV under drought conditions was mainly due to the indirect effects of LB and SD (Table 5). Stresses such as salinity and drought modify source-sink relationships and influence plant growth as well as adaptation to the stress [52]. The yield stability in plants subjected to stress requires a dynamic optimization of the source-sink relationships to maintain partitioning of photoassimilates and, at the same time, enable physiological and morphological adaptation responses. This optimization should be highly plastic and associated with stress severity, which will allow the survival of the plant under severe stress conditions, and proportion production stability compatible with the situation.

## Conclusions

Cacao progenies submitted to drought conditions showed different responses which depend on the plant organ evaluated. Reduction of leaf area, increase of root system and stem biomass were strategic mechanisms to the survival of cacao progenies under drought conditions. Root volume was the growth variable most influenced by the changes in soil water availability. Stem and root diameters as well as stem dry biomass were the growth variables with the greatest direct effects on the increase of root volume under drought conditions. These characters are indicated in screening of cacao progenies for drought tolerance.

## Supporting information

S1 File Mean values for growth characters of cacao progenies, obtained from complete diallel crosses, under control (predawn Ψ_WL_ between -0.1 and -0.5 MPa; Table A) and drought (Ψ_WL_ between -2.0 and -2.5 Mpa; Table B) condition. H = plant height (m); SD = stem diameter (mm); LN = leaves number per plant; TLA = total leaf area per plant (m^2^ x10^-4^); ILA = individual leaf area (m^2^ x 10^−2^); RB = root biomass (g); SB = stem dry biomass (g); LB = leaf dry biomass (g); TB = total dry biomass (g); R/S = root:shoot ratio; RL = root length (mm); RA = root system area (cm^2^); RD = root mean diameter for second order branches (mm); RV = root volume (cm^3^); RD[<1 mm] = fine roots length (cm cm^-3^); RD[1-2mm] = medium roots length (cm cm^-3^); RD[>2mm] = coarse roots length (cm cm^-3^).
(XLSX)
